# Comparative genomic and phenotypic description of *Escherichia ruysiae*: a newly identified member of the gut microbiome of the domestic dog

**DOI:** 10.3389/fmicb.2025.1558802

**Published:** 2025-04-01

**Authors:** Niokhor Dione, Kodjovi D. Mlaga, Siyi Liang, Guillaume Jospin, Zara Marfori, Nancy Alvarado, Elisa Scarsella, Ruchita Uttarwar, Holly H. Ganz

**Affiliations:** AnimalBiome, Oakland, CA, United States

**Keywords:** *Escherichia ruysiae*, canine microbiome, culturomics, whole genome sequencing, taxono-genomic

## Abstract

**Introduction:**

*Escherichia ruysiae* is a newly identified species within the *Escherichia genus*, yet its presence in domestic animals remains largely unexamined. This study characterizes four isolates detected for the first time in the domestic dog (Canis lupus familiaris), focusing on their phenotypic and genomic features.

**Methods:**

We used culturomic methods to isolate four *E. ruysiae* isolates that were initially identified as *Escherichia coli* using MALDI-TOF mass spectrometry. Whole-genome sequencing confirmed that the isolates belonged to *E. ruysiae*, not *E. coli*. Phenotypic characterization included enzymatic activity assays and antimicrobial susceptibility testing. Comparative genomic analyses were performed on these four isolates, along with 14 additional *E. ruysiae* and representative genomes from the five other *Escherichia species* in order to assess genetic diversity and functional gene distribution.

**Results and discussion:**

All strains exhibited similar enzymatic activities and resistance to clindamycin, erythromycin, and metronidazole. The pangenome analysis revealed that most missing gene orthologs are related to motility followed by metabolism, including synthetases, reductases, phosphatases, permeases, transferases, and epimerases, as well as structural genes like efflux pumps and transporters. Phylogroup typing using the ClermonTyping method identified two main groups within the *E. ruysiae* species, Clade III and IV. Typical virulence genes associated with *E. coli* are absent in these strains. The multiple approaches used in this study expand our understanding of the diverse aspects of the recently described species, *E. ruysiae*.

## Introduction

The gut microbiota of humans and animals comprises a diverse array of microbes that play critical roles in digestion, immune system function, and overall health. Advances in sequencing technologies, particularly metagenomics, have greatly enhanced our understanding of microbiome composition, enabling the discovery and characterization of new microorganisms and elucidating their roles in host biology. However, the identification of novel microorganisms remains largely dependent on existing databases of known species, limiting the scope of metagenomics. Only culture-based methods can recover live and previously unknown microorganisms (Ju et al., 2022).

Culturomics has emerged in the last decade as a powerful tool to culture and identify unknown bacteria in humans, animals, and the environment. The method consists of diversifying the culture media and conditions for the same sample by modifying parameters such as incubation time, temperature, pH, and atmosphere, or by adding inhibitory or growth factors such as antibiotics, promoters such as blood, and rumen fluid to culture low abundant and or fastidious populations, followed by the rapid identification by MALDI-TOF mass spectrometry. It enables the culture of enormous numbers of new microorganisms, including new species and genera associated with animal and human health and diseases, providing new perspectives on host–microbe interactions (Lagier et al., 2018, 2024).

We are conducting a major effort to identify and characterize the microbiome of domestic dogs. As part of this initiative we have identified over 400 *E. coli* isolates, through different steps: MALDI- TOF for initial screening, qPCR for species confirmation, multiplex PCR for phylogrouping (Dogan et al., 2020) and Whole Genome Sequencing (WGS) to classify serotype and pathogenicity groups. Following WGS we discovered a subset of isolates with a distinct core genome compared to the rest. Further analysis using the Average Nucleotide Identity (ANI) confirmed that these isolates represent a different species within the *Escherichia* genus: *E. ruysiae*.

Non-*Escherichia coli* encompasses cryptic *clades I, II, III*, and *IV*, and *E. ruysiae* is the latest species described within the *Escherichia genus*. This genus also includes four other species: *Escherichia coli*, *Escherichia albertii*, *Escherichia fergusonii*, and *Escherichia marmotae*. The first *E. ruysiae* strain was isolated from a fecal sample collected in the Netherlands from a human (*Homo sapiens*) who may have acquired the strain while traveling internationally for a month in Asia and developed traveler’s diarrhea (van der Putten et al., 2021). So far, its dissemination and potential role in human and animal health is not well characterized. *Escherichia ruysiae* was subsequently isolated from a fecal sample collected from a Lohmann Brown layer hen (*Gallus gallus domesticus*) on a farm in England (Thomson et al., 2022) and from a fecal sample collected from an urban dwelling large-billed crow (*Corvus macrorhynchos*) in Japan (Sakaguchi et al., 2023).

In this context of wide dissemination, it is important to understand the genomic features that underline their spread, mechanisms of acquisition of antimicrobial and virulence genes, and the genomic characteristics that support their evolution. Here we describe the phenotypic and genomic features of these new isolates from the domestic dog and perform a comparative genomics analysis with representative species of other *Escherichia* available in the public repository NCBI.

## Materials and methods

### Sample information, culture, and identification

The stool sample used in this study was collected from a healthy spayed female 9-year-old Shepherd-Husky mix living in Oakland, California, United States. After collection, the samples were transported to the laboratory and homogenized using BIOME-Preserve AS-930 (Anaerobe Systems, Morgan Hill, CA) in a one-to-one ratio. This sample was processed as part of our culturomics efforts to characterize the microbiome of companion pets by culturing, isolating, identifying, and characterizing bacteria. The *E. ruysiae* strains that are the focus of this study were isolated following culturomics protocols as previously described by Lagier et al. (2012). Briefly, 100 μL of the aliquoted sample was serially diluted (10^−1^–10^−10^) in Dilution Blank AS-9183 (Anaerobe Systems, Morgan Hill, CA) then 70 μL of each dilution was inoculated into a MacConkey agar plate (Becton Dickinson, Franklin Lakes, NJ 07417), and incubated aerobically at 37°C for 24 h. Following standard procedures, grown colonies were first identified using the MALDI–TOF mass spectrometer (MS). A scrapped colony is deposited on a disposable MALDI-TOF MS target plate (Bruker Daltonics, Bremen, Germany) and covered first with 2 μL of 70% formic acid solution and dried before being covered by 2 μL of a MALDI matrix solution (saturated α-cyano acid-4- hydroxycinnamic in 50% acetonitrile, 47.5% HPLC water, and 2.5% trifluoroacetic acid). After drying, analysis was performed using MALDI Biotyper smart System RUO microflex LT/SH smart with the BIDAL database v12 (Bruker Daltonics, Bremen, Germany). A triplex PCR was carried out using the Clermont typing method (Clermont et al., 2000) followed by whole genome sequencing using the Pacific Biosciences Sequel IIe.

### Phenotypic characterization

The newly isolated bacterial strains were identified first using the MALDI-TOF MS instrument (Bruker Daltonics, Bremen, Germany) as per a previous study (Asare et al., 2023). Additionally, bacterial characterization was conducted utilizing the Gram staining technique (Sigma Aldrich, MA, United States), while motility was observed using the hanging drop method under a light microscope (Accu-Scope, NY, United States), as described in earlier studies (Jain et al., 2020). The catalase, oxidase (bioMérieux, Marcy-l’Étoile, France), and biochemical tests API ZYM (bioMérieux, Marcy-l’Étoile, France) were conducted to study the bacterial phenotypic characteristics. Furthermore, these strains were also visualized to characterize their morphology and structure. Fixed bacteria were placed on a 400 mesh Formvar-coated copper grid that had been previously subjected to glow discharge (Electron Microscopy Sciences, PA, United States). Following a settlement period of 2 min for the bacteria, the grids were thoroughly rinsed with UranyLess EM Stain (Electron Microscopy Sciences, PA, United States) and the remaining UranyLess EM Stain was removed. Once dried, the grids were imaged at a voltage of 120 kV using a Tecnai 12 (Thermo Fisher Scientific, Waltham, MA, United States) Transmission Electron Microscope (TEM), located in the Electron Microscope Lab at the University of California, Berkeley.

### Antimicrobial susceptibility testing

We used the disk diffusion method to screen the antimicrobial susceptibility of all bacteria isolates. We screened the *E. ruysiae* isolates against 11 antibiotic drugs, sourced from Thermo Fisher Scientific (Waltham, MA, United States), using the following disks: Metronidazole 50 μg, Cefpodoxime 10 μg, Enrofloxacin 5 μg, Erythromycin 15 μg, Amoxicillin/Clavulanic acid 30 μg, Tetracycline 30 μg, Sulfamethoxazole/Trimethoprim 25 μg, Doxycycline 30 μg, Clindamycin 2 μg, Gentamicin 10 μg, and Chloramphenicol 30 μg. We inoculated *E. ruysiae* isolates onto Columbia agar plates and incubated them at 37°C for 24 h. Pure colonies were introduced into Dilution Blank AS-9183 (Anaerobe Systems, Morgan Hill, CA) to prepare a 0.5 McFarland bacterial suspension. This suspension was then used to inoculate Mueller Hinton agar plates Becton Dickinson BBL™ (Franklin Lakes, NJ, United States) using a wet swab following the SLCI guidelines. Antibiotic disks were then placed on the surface of the plates with the aid of a Sensi-disk self-tamping 8-place dispenser Becton Dickinson BBL^™^ (Franklin Lakes, NJ, United States). The plates were incubated at 37°C for 24 h and inhibition zones were read by BIOMIC V3 (Giles Scientific in Santa Barbara, CA, United States). Each isolate was tested in triplicate.

### DNA extraction and whole genome sequencing

We extracted DNA using the ZymoBIOMICS™ DNA Miniprep Kit for bacterial cells (Zymo Research, Irvine, CA, United States). The samples were first prepared by inoculating a bacterial colony into a 2 mL microcentrifuge tube containing 1,000 μL phosphate buffered saline (PBS) (Thermo Fisher Scientific, Waltham, MA, United States) and centrifuging at 5,000 RPM for 10 min. The PBS was removed without dislodging the pellet. Twenty μl of 20 mg/mL lysozyme & 750 μL ZymoBIOMICS lysis solution was then added and incubated at 37°C for 40 min. The samples were then transferred to ZR BashingBead™ Lysis tubes and secured in a Vortex-Genie® 2 mixer fitted with a 2 mL tube holder adapter and vortexed at full speed for 40 min. DNA concentration was measured using a Qubit 4 Fluorometer (Thermo Fisher Scientific, Waltham, MA, United States). Genomic DNA was sheared to a 7–12 kb fragment length on a Megaruptor 3 (Diagenode LLC., Denville, NJ, United States). The quality of the DNA was examined on a 2% agarose gel. The sequencing library was prepared following the manufacturer’s protocol using the SMRTbell prep kit 3.0 and and the isolates were barcoded using the SMRTbell barcoded adapter plate 3.0 (Pacific Biosciences, Menlo Park, CA, United States). We loaded 115 μL of the pooled library onto a 96-well sample plate and prepared it for a sequencing run on a Sequel IIe sequencer using Binding kit 3.2, Sequencing kit 2.0, and SMRT Cell 8 M (Pacific Biosciences, Menlo Park, CA, United States). The library was sequenced using a 15-h movie. HiFi data was obtained from the raw subreads using the circular consensus sequencing process.

### Comparative genomics

#### Genome assembly and annotation

All sequencing reads were screened for quality control using Fastqc v0.12.1 (Andrews, 2010). The sequencing reads were filtered with a Phred score > 30, renamed by sample ID, and assembled using Canu assembler v2.3 (Koren, et al. 2017) with default parameters. The final scaffold files produced were screened for level of contamination and purity using CheckM2 v1.0.2 (Chklovski et al., 2023) and taxonomically classified using GTDB-Tk v2.4.0 (RefDB version r220) (Chaumeil et al., 2022). We annotated the four genomes using Prokka v1.14.6 (Seemann, 2014) with an *e*-value set to 0.00001, a minimum coverage of 95%, using the specific database of the genus *Escherichia* (Supplementary Table S1). In addition, 14 genomes of *E. ruysiae* (Supplementary Table S2) and one representative genome of each other species in the *Escherichia* genus (Supplementary Table S3) were also retrieved from the NCBI Refseq database for comparative analysis purposes and re-annotated using the same methods.

### Isolate sequence typing, serogrouping and phylogrouping

We performed an *in silico* Multi-Locus Sequence Type (MLST) analysis using MLST package, version 2.23.0,^1^ adapted from https://pubmlst.org (Jolley and Maiden, 2010) using these seven housekeeping genes: *adk, fumC, gyrB, icd, mdh, purA,* and *recA*. *Escherichia ruysiae* species are part of the cryptic clades I, II, III, and IV (Walk, 2015). We used the software ClermonTyping version 1.4.0 (Beghain et al., 2018) to determine the phylogroups of all the genomes.

#### Pangenome analysis

We performed a pangenome analysis, searching for clusters of ortholog genes using get_homologues v22082022 (Contreras-Moreira and Vinuesa, 2013; Vinuesa and Contreras-Moreira, 2015). We used two approaches to search for ortholog genes, namely OrthoMCL v1.4 (OMCL) (Li et al., 2003) and Clusters of Orthologous Genes (COG) triangles v2.1 (Kurtz et al., 2004; Kristensen et al., 2010) clustering algorithm. Sequence similarity requires an equal Pfam domain, and all clusters detected were reported. The processes were performed locally using default parameters. First we calculated the Average Nucleotide Identity (ANI) and the Percentage of Conserved Proteins (POCPs) of the *Escherichia* genus in order to determine the level of delineation and to determine the relatedness of the newly isolated four strains to publicly available *E. ruysiae* genomes. Then in order to support the ANI-based species delineation, we constructed a core genome midpoint-rooted Maximum Likelihood phylogenetic tree of 23 species, including 18 *E. ruysiae* strains and five representative genomes from other *Escherichia* species, using 1,000 bootstrap replicates. We computed the core and pan-genomes, including their evolution, composition, and the parsimony tree of *E. ruysiae* strains using the intersection of both OMCL and COG approaches. In addition, we queried the pangenome to determine accessory (shell) and specific (cloud) ortholog genes of our strains versus others.

#### Phylogenetic reconstruction, genomics markers, resistome and virulome

We performed a whole and a core genome alignment of all 18 genomes of *E. ruysiae* using *scapper*,^2^ a simple and fast SNP alignment tool that uses mummer v4.0.0 (Kurtz et al., 2004) and Trimal v1.4.rev22 (Capella-Gutiérrez et al., 2009). We reconstructed the phylogenetic evolution of the core genome using FastTree v2.1 (Price et al., 2009). We screened the resistome profile by mapping the full genome of *E. ruysiae* to various resistance gene databases including NCBI (Feldgarden et al., 2019), and ARGANNOT (Gupta et al., 2014), ResFinder (Zankari et al., 2012), CARD (McArthur et al., 2013) and MEGARes 2.0 (Bonin et al., 2023) to determine genes associated to antimicrobial resistance using ABRicate software.^3^

## Results

### Culture, isolation, and phenotypic characterization

**Figure 1:**
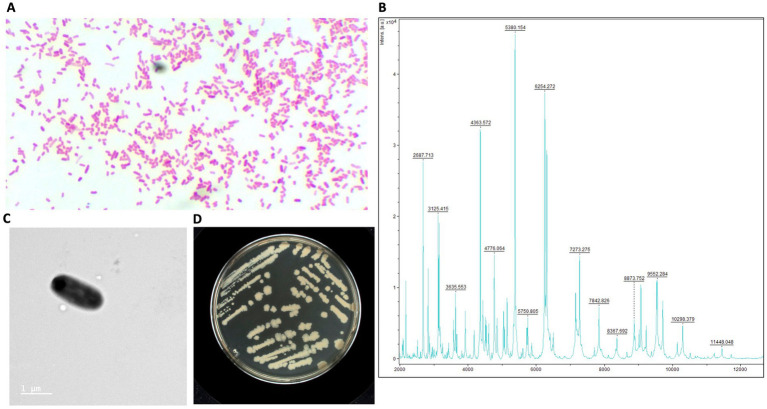
Structural and phenotypic characterization of *E. ruysiae* (strain AB135). **(A)** Gram stain of colonies under 100X light microscopy, **(B)** MALDI-TOF spectrum, **(C)** Electronic microscopy with rod-shape bacterium, **(D)** Colony morphology and color on LB agar.

Strains AB134, AB135, AB136, and AB137 were isolated in July 2022 after 24 h of incubation on a MacConkey Agar plate (BD) following the culturomics process. A pure colony of each isolate was tentatively identified using MALDI-TOF mass spectrometry (MBT Sirius One RUO; Bruker Daltonics, Bremen, Germany). The four strains were identified as *E. coli* with a score of 1.8 which is the identification threshold at the genus level. This result was due to the absence of a reference spectrum of *E. ruysiae* in the Bruker BIDAL database v12 used in the analysis. The triplex PCR also did not allow clear identification of *E. ruysiae* We performed whole genome sequencing using PacBio sequencing technology and identified the strains *AB134, AB135, AB136*, *AB137* as *E. ruysiae* with Gtdbtk based on the Average Nucleic Identity (ANI), which were 98.94, 98.93, 98.92, and 97.1%, respectively. Gram staining performed using the Sigma Aldrich Gram Staining Kit (St. Louis MI, United States) showed Gram-negative bacilli rod-shaped (Figure 1A). After identification, we performed a protein extraction as recommended by Bruker to generate a reference spectrum (Figure 1B) using 12 individual colonies of strains AB134 grown on in-house LB agar (Waltham, MA, United States) made from a dehydrated medium.

The rod-shaped bacilli form was confirmed by electron microscopy, where strain *AB134* exhibited a mean length of 1.8 μm and width of 0.6 μm (Figure 1C). When grown on MacConkey agar plates, *E. ruysiae* exhibit irregular dark white colonies with a diameter of about 0.3–0.8 mm (Figure 1D). Evaluation of enzymatic activity of *E. ruysiae* strains AB134, AB135, AB136, and AB137 using API ZYM revealed that all strains exhibited similar enzymatic activities with positive reactions observed with alkaline phosphatase, leucine arylamidase, acid phosphatase, naphthol-AS-BI-phosphohydrolase α-galactosidase, ß-galactosidase, and α-glucosidase. All *E. ruysiae* show negative activity on the other hand with the following enzymes: esterase (C4), esterase lipase (C8), lipase (C14), valine arylamidase, cystine arylamidase, trypsin, α-chymotrypsin, α-galactosidase, ß-glucuronidase, ß-glucosidase, N-acetyl-ß-glucosaminidase, α-mannosidase, and α-fucosidase (Table 1). The API 20E profile evaluating amino acids, salts, activity, and carbon source shows Positive activity for 2-nitrophenyl-ȕD galactopyranoside, L-ornithine, sodium pyruvate, D-glucose, D-mannitol, D-sorbitol, L-rhamnose, and L-arabiose, and negative activity for L-arginine, L-lysine, trisodium citrate, sodium thiosulfate, urea, L-tryptophan, gelatin (bovine origin), D-saccharose, D-melibiose, and amygdalin (Table 1).

**Table 1:** Structure, morphology, and phenotypic characteristics of *E. ruysiae*, strains AB134, AB135, AB136, and AB137. The type strain OPT1704T is listed here to indicate that it was not characterized phenotypically.

Substrates tested	*E. ruysiae AB134*	*E. ruysiae AB135*	*E. ruysiae AB136*	*E. ruysiae AB137*	*E. ruysiae OPT1704T*
Basic characteristics					
Oxygen requirement	Facultative anaerobe	Facultative anaerobe	Facultative anaerobe	Facultative anaerobe	Facultative anaerobe
Gram stain	−	−	−	−	
Motility	Non-motile	Non-motile	Non-motile	Non-motile	
Endospore formation	−	−	−	−	
Enzyme					
Alkaline phosphatase	+	+	+	+	NA
Esterase (C 4)	−	−	−	−	NA
Esterase Lipase (C 8)	−	−	−	−	NA
Lipase (C 14)	−	−	−	−	NA
Leucine arylamidase	+	+	+	+	NA
Valine arylamidase	−	−	−	−	NA
Cystine arylamidase	−	−	−	−	NA
Trypsin	−	−	−	−	NA
α-chymotrypsin	−	−	−	−	NA
Acid phosphatase	+	+	+	+	NA
Naphthol-AS-BI-phosphohydrolase α-galactosidase	+	+	+	+	NA
α-galactosidase	−	−	−	−	NA
ß-galactosidase	+	+	+	+	NA
ß-glucuronidase	−	−	−	−	NA
α-glucosidase	+	+	+	+	NA
ß-glucosidase	−	−	−	−	NA
N-acetyl-ß-glucosaminidase	−	−	−	−	NA
α-mannosidase	−	−	−	−	NA
α-fucosidase	−	−	−	−	NA
Amino acids/salts/carbon source					
2-Nitrophenyl-ȕD galactopyranoside	+	+	+	+	NA
L-Arginine	−	−	−	−	NA
L-Lysine	−	−	−	−	NA
L-Ornithine	+	+	+	+	NA
Trisodium citrate	−	−	−	−	NA
Sodium thiosulfate	−	−	−	−	NA
Urea	−	−	−	−	NA
L-Tryptophan	−	−	−	−	NA
L-Tryptophan	+	+	+	+	NA
Sodium pyruvate	+	+	+	+	NA
Gelatin (bovine origin)	−	−	−	−	NA
D-Glucose	+	+	+	+	NA
D-Mannitol	+	+	+	+	NA
Inositol	−	−	−	−	NA
D-Sorbitol	+	+	+	+	NA
L-Rhamnose	+	+	+	+	NA
D-Saccharose	−	−	−	−	NA
D-Melibiose	−	−	−	−	NA
Amygdalin	−	−	−	−	NA
L-Arabiose	+	+	+	+	NA
Origin	Canine gut	Canine gut	Canine gut	Canine gut	NA

**Figure 2:**
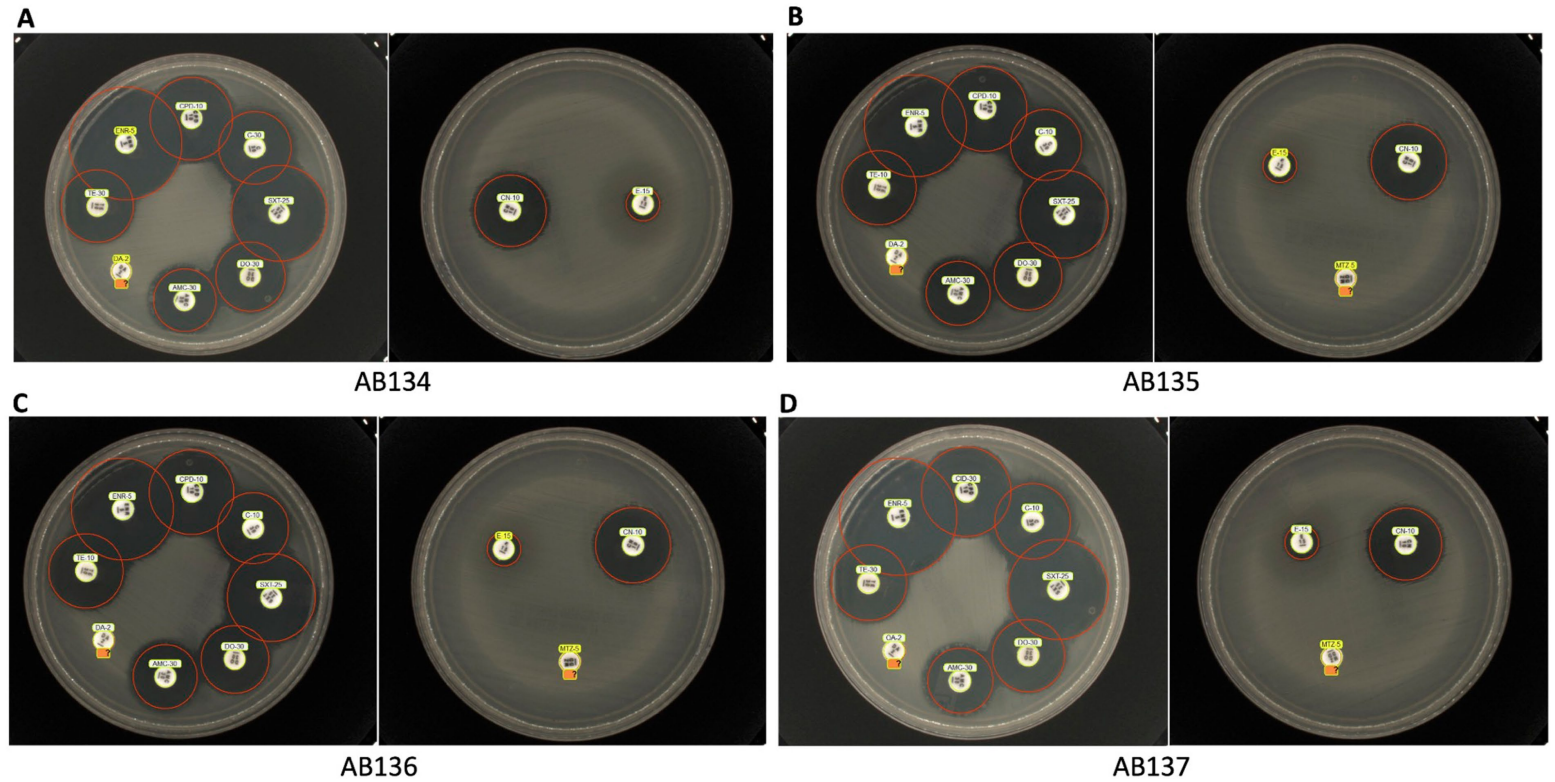
Antimicrobial susceptibility testing of *E. ruysiae* isolates. The figure shows results for multiple isolates AB134 **(A)**, AB135 **(B)**, AB136 **(C)**, and AB137 **(D)** against trimethoprim-sulfamethoxazole, cefpodoxime, enrofloxacin, chloramphenicol, tetracycline, doxycycline, amoxicillin-clavulanate, gentamicin, clindamycin, erythromycin, and metronidazole (with the exception that metronidazole susceptibility was not tested for AB134).

From the disk diffusion method, these *E. ruysiae* strains exhibited susceptibility to cefpodoxime, enrofloxacin, chloramphenicol, tetracycline, doxycycline, trimethoprim-sulfamethoxazole, and gentamicin. The strains exhibited resistance to clindamycin, erythromycin, and metronidazole, while showing an intermediate response for amoxicillin-clavulanate (Table 2; Figure 2).

**Table 2:** Antimicrobial Susceptibility testing results of *E. rusyiae* strains AB134, AB135, AB135, AB136 compared to *E. ruysiae* strains S1-IND-07-A (Campos-Madueno et al., 2023).

Antibiotics	ZD (mm) value for new isolates				MIC value (mg/mL)
	AB134	AB135	AB136	AB137	S1-IND-07-A
Cefpodoxime	25(S)	25(S)	22(S)	26(S)	2(S)
Enrofloxacin	34(S)	30(S)	34(S)	34(S)	NA
Chloramphenicol	22(S)	21(S)	22(S)	22(S)	NA
Tetracycline	22(S)	22(S)	22(S)	22(S)	NA
Trimethoprim-sulfamethoxazole	29(S)	26(S)	28(S)	29(S)	>4/76(S)
Clindamycin	6(R)	6(R)	6(R)	6(R)	NA
Doxycycline	21(S)	20(S)	22(S)	21(S)	16
Amoxicillin-clavulanate	19(S)	19(S)	19(S)	19(S)	NA
Erythromycin	10(R)	10	10(R)	10(R)	NA
Gentamicin	21(S)	22(S)	21	21(S)	≤1(S)
Metronidazole	6(R)	6(R)	6(R)	6(R)	NA

### Genome features, taxono-genomics, and genomic description

**Figure 3:**
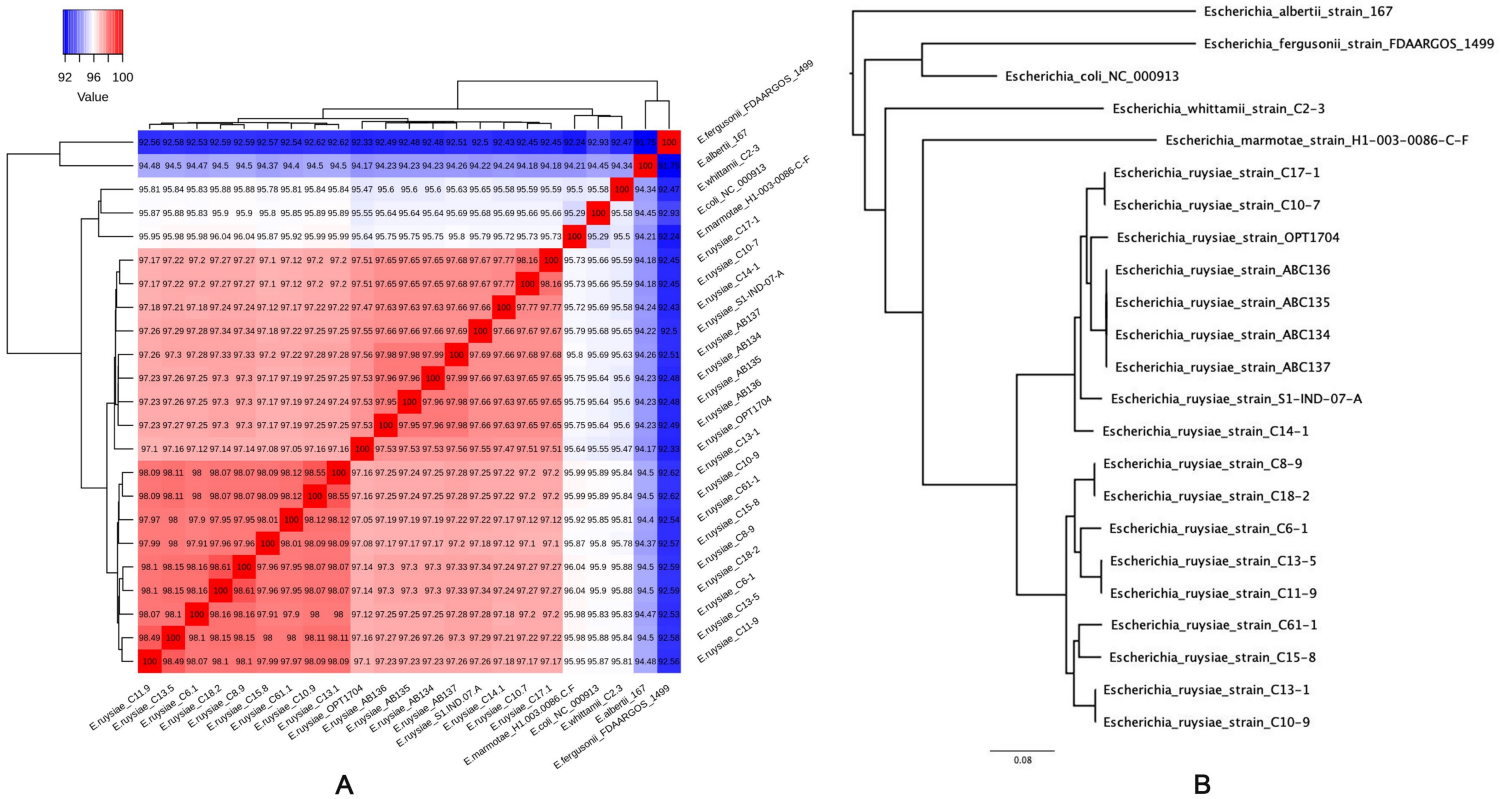
Average Nucleotide Identity (ANI) of *E. ruysiae*, *E. coli*, *E. marmotae*, *E. whittami*, *E. albertii*, and *E. fergusonii.* **(A)** Heatmap with overall ANI of all the available genomes of *E. ruysiae.* **(B)** Midpoint rooting maximum likelihood tree based on core genome alignment (RAxML best tree).

Genomes of the four strains were submitted and referenced in the NCBI database with the respective accession numbers: *E. ruysiae* strain AB134 (JAVIWR000000000), *E. ruysiae* strain AB135 (JAVIWS000000000), *E. ruysiae strain* AB136 (JAVIWT000000000), and *E. ruysiae strain* AB137 (JAVIWU000000000). The genomes were assembled, annotated, and genome features described (Table 3). All genomes are circularized in one contig of 4.5 Mb average size and 95 kb of circularized plasmid. We checked the quality of the assemblies with CheckM which indicated 100% completeness with an average GC% of 50.6%. Genomic accession numbers, bioproject number, and links for the four new *E. ruysiae* genomes are provided in Supplementary material (Supplementary Table S1). The genomes were taxonomically classified using Gtdbtk and the results indicated that all four genomes are identified as *E. ruysiae* with a minimum of 97.1% of Average Nucleotide Identity (ANI) and 0.95 of Alignment Fraction (AF) with *E. ruysiae* GCF_902498915.1 (Supplementary material). We computed the overall ANI of all the available genomes of *E. ruysiae* including reference genomes of *E. coli*, *E. marmotae*, *E albertii*, *E. fergusonii*, and *E. whittamii* using the OMCL ortholog finding algorithm in get_homologes with default parameters. The heatmap (Figure 3A) indicates that all the *E. ruysiae* (red square) forms a distinct cluster, separate from other *Escherichia* species (white to blue). Additionally, *E. ruysiae* are clustered in two distinct groups, with a minimum of 97.47% and a maximum of 98.6% ANI. The four strains sequenced in this study AB134, AB135, AB136, and AB137 belong to the same group with ANI ranging from 98.94 to 97.6%. The closest related species is *E. coli* NC000913 with 95% on average and the most distant species is *E. fergusonii strain* FDAARGOS 1499 (ANI: 92.41–92.51%). The core genome midpoint-rooted Maximum Likelihood tree indicates that all *E. ruysiae* strains are closely related, forming two distinct clusters separate from the other species (Figure 3B). In contrast, *E. coli*, *E. marmotae*, *E. whittamii*, *E. albertii*, and *E. fergusonii* exhibit more distant relationships.

**Table 3:** *E. ruysiae* genomes features from genome assembly and annotation.

Isolate ID	Contigs	Genome size*	N50	Susp.Plasmids	Completeness	CDS	genes	Repeat regions	rRNA	tRNA	mRNA	GC%
AB134	2	4,635,588	4,540,385	95,207	100	4,252	4,367	3	22	92	1	50.67
AB135	2	4,632,218	4,537,266	94,952	100	4,253	4,368	3	22	92	1	50.68
AS136	2	4,635,326	4,540,375	94,951	100	4,248	4,363	3	22	92	1	50.67
AB137	2	4,635,425	4,540,363	95,062	100	4,261	4,376	3	22	92	1	50.67

### Pangenome description of *E. ruysiae* species

We clustered the genes from all genomes into ortholog genes using get homolog. From the 18 publicly available genomes (Supplementary Table S2), we identified 6,714 total genes from both OMCL (173 specifics) and COG (186 specifics) with 3,231 genes included in the hard core genes (present in all 18 genomes) and 3,330 genes in the soft core (at least 17 genomes possess these genes). One thousand four hundred eighty-five genes were shared across 10 to 95% of the genomes (accessory or shell genes) and 1899 genes are specific to each genome (cloud genes). The hard core genome estimation using the Tettelin fit converged with residual standard error = 100.76 and fitting value from 4,105 genes to 3,284 following the equation:


$$C\left(g\right)=3284+1324*e^{\left(-g/2.10\right)}$$

**Figure 4:**
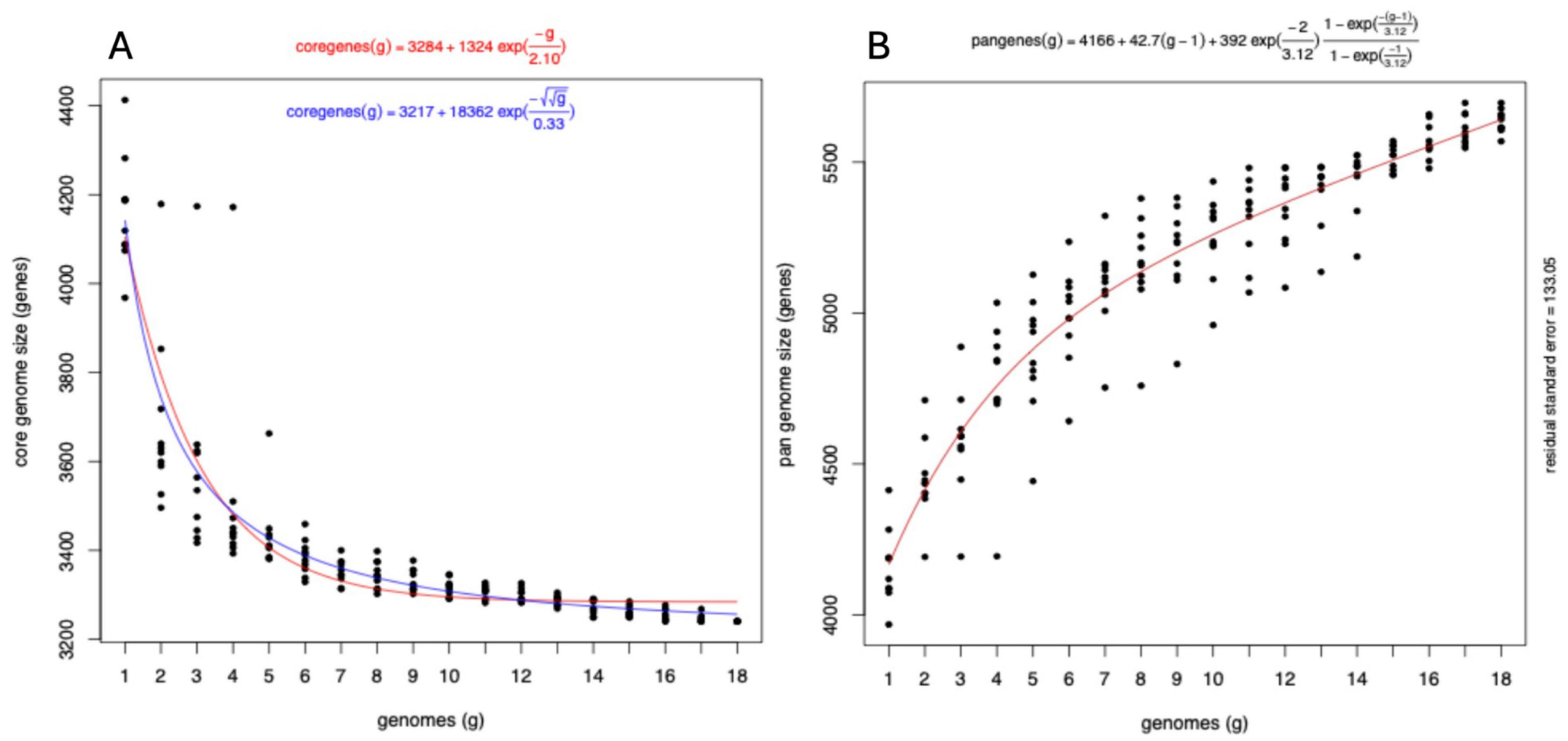
Evolution of the pangenome of *E. ruysiae*. **(A)** Hard core genome estimation using the Tettelin fit with residual error estimated using the Willenbrock method. **(B)** Pangenome fit is consistent with an open pangenome.

Using the Willenbrock method, the residual error was estimated to be 95.08 with the fitting value from 4,142 to 3,256 following the equation (Figure 4A):


$$C\left(g\right)=3217+18362*e^{\left(-\sqrt{\{\left(\}\sqrt{g}\right)}/0.33\right)}$$

Tettelin fitting methods revealed that the variation in gene numbers from the 16th genome onwards is negligible, with only four additional genes identified after the 11th genome. This suggests that the hard core genome has reached a stable state and undergoes minimal variation. The pan-genome also fit converged with the estimated residual error of 133.05 following the curve equation of


$$\begin{array}{l} P\left(g\right)=4166+42.7*\left(g-1\right)+392*e^{\left(-2/3.12\right)}*\\ \left(1-e^{\left(-\left(g-1\right)/3.12\right)}\right)/\left(1-e^{\left(-1/3.12\right)}\right) \end{array}$$

This equation shows the pangenome size keeps increasing indefinitely when adding a new genome, hence is interpreted as representing an open pangenome (Figure 4B). The four genomes sequenced for this study: AB134, AB135, AB136, and AB137 have altogether 147 unique groups of genes including accessory and unique genes, and are missing 923 groups of genes. The unique group of genes includes genes associated with virulence such as *invasin* (group 1,338), type II secretion system protein J, G, F, and E (*xcpW, epsG, epsF*, and *epsE* respectively), flagella-associated system genes (*flgL, flgH, flgG, flgH, flgE, flgC, tagD, fliI, fliE, vnfA, fliP, flihB*), *flagellin*, and *adhesin BmaC*. we also identified groups of genes associated with evolution mechanisms that include transposon and phage transfer and acquisition [Insertion sequence family transposase (*ISSen1* and *ISEc48* among many others), *endonuclease* and *endoribonuclease, recombinase, prophage integrase (IntS)*]. The majority of missing groups of genes are motility-associated genes and metabolism-associated genes including synthetases, reductases, phosphatases, permeases, transferases, and epimerases, as well as structural-related genes such as efflux pumps and transporters (Supplementary Figure S1).

### Phylogenetic analysis and evolution of *E. ruysiae* species

**Figure 5:**
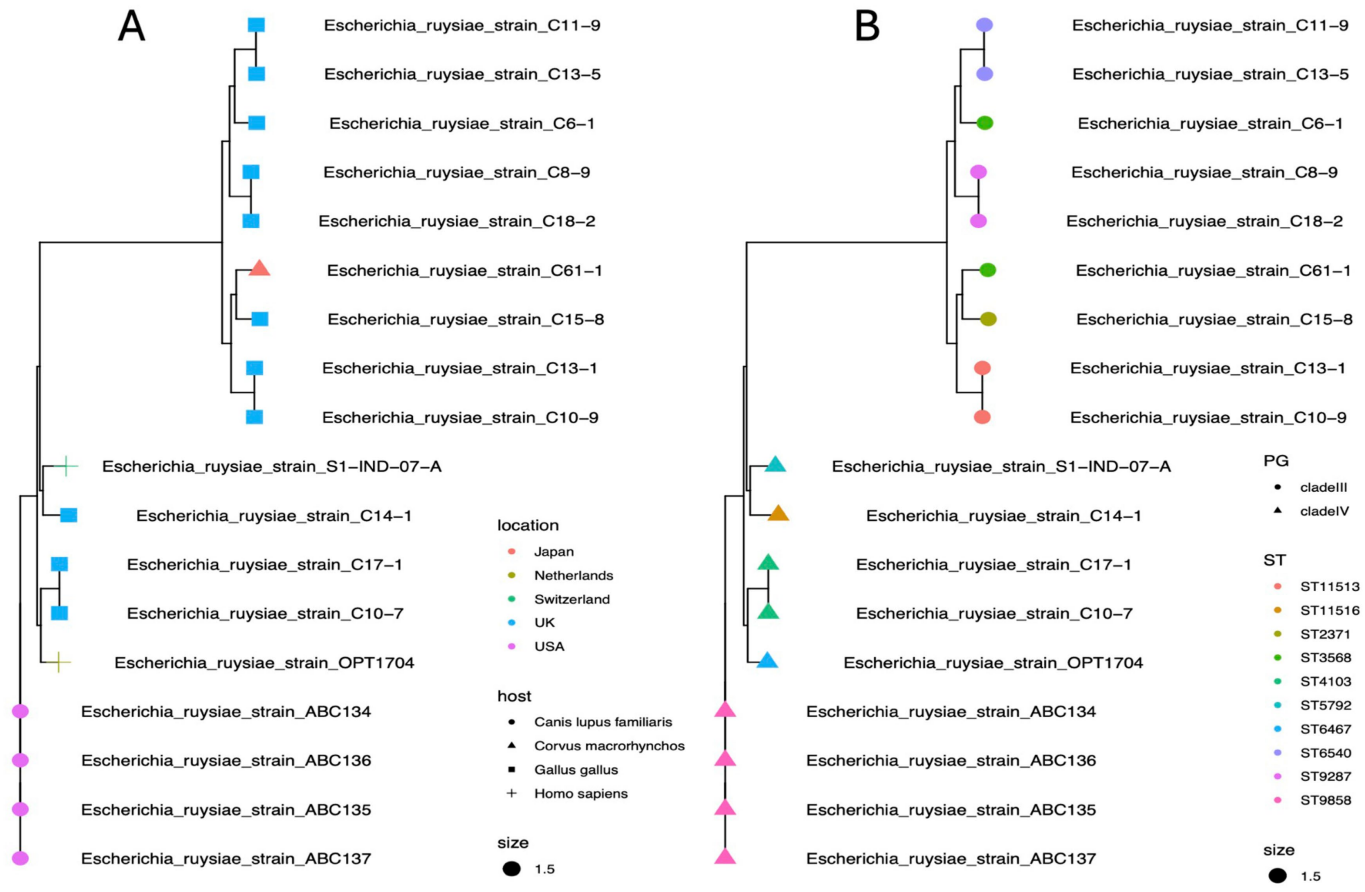
Maximum likelihood phylogenetic reconstruction of *E. ruysiae* using core genome alignment. **(A)** Tree showing diversity by geographical location (tip colors) and host (tip shapes). **(B)** Tree showing diversity by phylogroups (tip shapes) and sequence types (ST, tip colors).

Phylogroup typing using the ClermonTyping method showed that there are two main groups within the *E. ruysiea* species, namely *Clade III* characterized by genes *trpA, trpBA*, and *chuIII* and *Clade IV* characterized by the genes *trpBA, chuIV*, and facultatively *trpAgpC*. All typical genes for *E. coli* typing were absent (*arpA, chuA, yjaA,* and *TspE4.C2*). All genomes sequenced for this study belong to *Clade IV*. The topology of the estimated phylogenetic tree, based on the core genome, shows two distinct clades positioned on separate branches with a midpoint rooting layout (Figure 5A). The phylogenetic distribution and the clade type are neither geographically- nor host-dependent. Strains isolated from chicken occur in both *Clades III* and *IV* while the human and dog strains isolated to date only occur in *Clade IV* (Figure 5B). All *Clade III* strains including the sequencing type (ST): *ST6540, ST3568, ST9287, ST2371,* and *ST11513* are located on the top branch, and the *Clade IV* with ST: *ST6467, ST11516, ST4103, ST5792, ST9858* on the bottom branch (Figure 5B). Analysis of the 18 *E. ruysiae* isolates suggests that phylogenetic distribution and clade type are not strongly associated with geography or host species. However, this pattern likely reflects the limited number of isolates identified to date, given that *E. ruysiae* is a recently described cryptic species. Expanding efforts to isolate *E. ruysiae* from diverse hosts across a broad geographical range may provide deeper insights into the evolution of these clades and their adaptations to different environments.

### Resistome and virulome description

**Figure 6:**
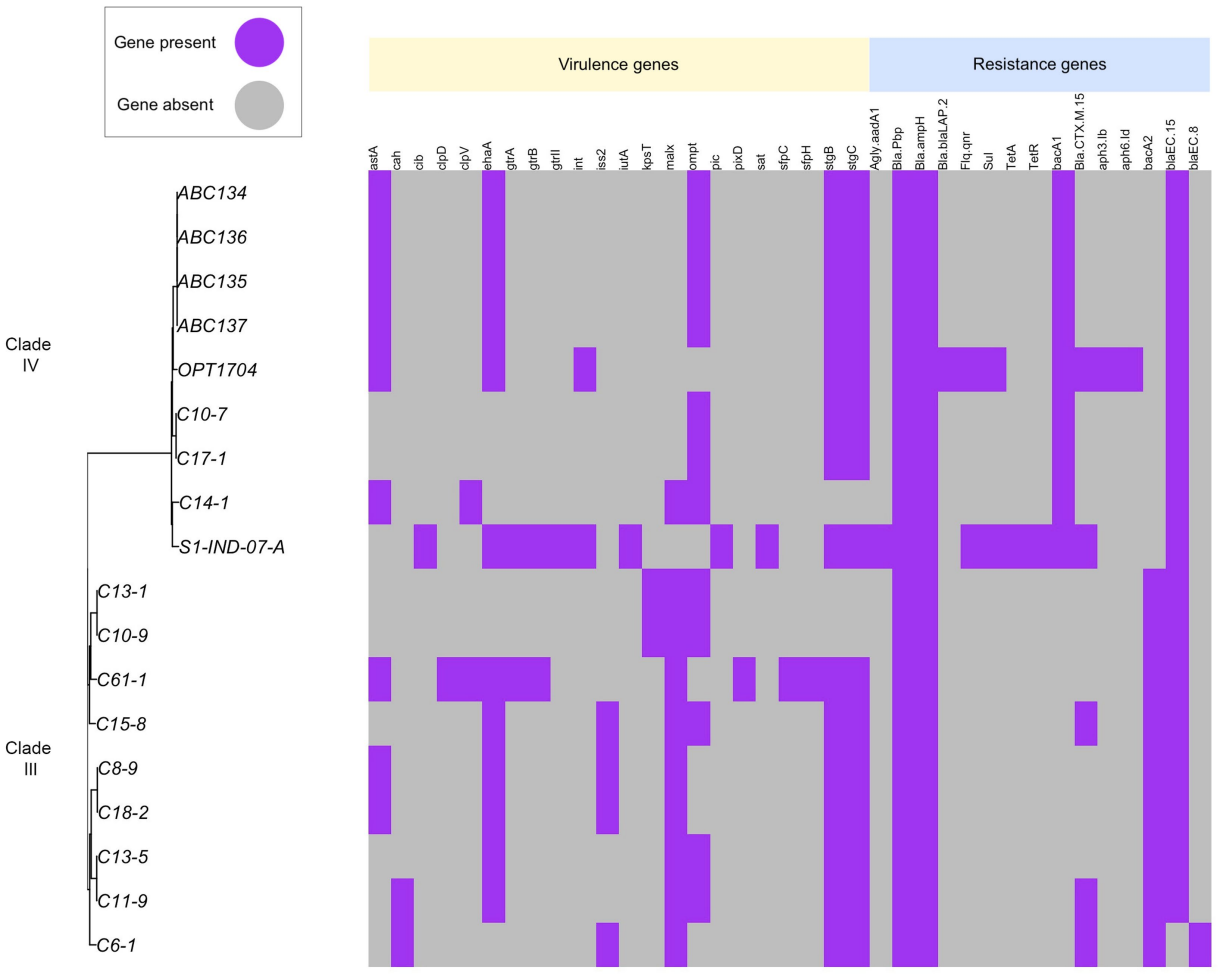
Distribution of virulence genes and antimicrobial resistance genes across *E. ruysiae*. Strain C6-1 isolated from chicken feces possesses some virulence genes but lacks common resistance genes, suggesting potential for infection but possibly treatable with a relatively wide range of antibiotics. Strains from humans (OPT1704, S1-IND-07-A) harbor both virulence genes and multiple resistance genes, indicating a potentially high risk for antibiotic-resistant infections. Aminoglycoside resistance genes Agly-aadA (aminoglycoside-3-adenyltransferase) and penicillin-binding protein (Bla-Pbinding protein) were found in all strains. Beta-lactamase BlaCTX-14, Bla-CTX-8, and Bla-CTX-15 were identified in both clades.

We identified genes associated with antimicrobial resistance in all genomes. Aminoglycoside resistance genes *Agly-aadA (aminoglycoside-3-adenyltransferase)* and penicillin-binding protein (*Bla-Pbinding protein*) occurred in all strains. Beta-lactamase *BlaCTX-14, Bla-CTX-8*, and *Bla-CTX-15* can be identified among both clades. The strain *C6-1* isolated from chicken feces possesses some virulence genes but lacks common resistance genes, suggesting potential for infection but possibly treatable with a wider range of antibiotics. Human strains (*OPT1704, S1-IND-07-A*) harbor both virulence genes and multiple resistance genes, indicating a potential risk for antibiotic-resistant infections (Figure 6).

## Discussion

The first successful isolation and culturing of *E. ruysiae* from the fecal microbiome of a healthy domestic dog in western United States expands our understanding of its host range and geographic distribution. The reported isolation of *E. ruysiae* from a diversity of animal hosts (a human traveler (van der Putten et al., 2021), an urban wild crow (Sakaguchi et al., 2023), a domestic chicken (Thomson et al., 2022), and now a domestic dog) on three different continents (Europe, Asia, North America) underscores the adaptability of this species and suggests a global distribution. Using culturomic and genomic techniques, we gained new insights into the previously elusive growth requirements and metabolic capabilities of *E. ruysiae*, findings that may help facilitate future isolation efforts.

All strains isolated in this study grew on MacConkey Agar plates, forming colonies with distinct size and color after 18–24 h aerobic incubation at 37°C, following the culturomics process (Lagier et al., 2018). Previous studies reported that *E. ruysiae* forms circular, gray-white colonies on a Columbia sheep blood agar plates after overnight incubation at 37°C (Van Der Putten et al., 2021). MALDI-TOF was unable to distinguish between *E. coli* and *E. ruysiae* because the spectral profile for *E. ruysiae* had not yet been included in the databases of both the MALDI-TOF (Bruker Daltonics, Bremen, Germany) and VITEK2 (BioMérieux, Marcy-l’Étoile, France) systems (van der Putten et al., 2021). The differences between these species became apparent only when we compared the genomes of the isolates with those of *E. coli*. Whole genome sequencing, which confirmed the identification of *E. ruysiae*, remains the gold standard for accurate taxonomic identification, and highlights the limitations of MALDI-TOF when using an incomplete database. Nonetheless the MALDI-TOF remains an excellent identification tool, particularly for well characterized species and is invaluable for culturing-based microbiome analysis (Demir and Hazırolan, 2024).

A detailed phenotypic characterization of *E. ruysiae* strains revealed distinctive features that may contribute to the bacterium’s ecological niche. Analysis of biochemical properties and antibiotic susceptibility profiles highlighted the species’ phenotypic diversity. Notably, reduced motility and probable loss of flagella were consistent with the initial description of *E. ruysiae* (van der Putten et al., 2021) and may represent an adaptation to a commensal lifestyle. Similar loss of flagellar motility was observed in an experimental evolution study where non-symbiotic *E. coli* evolved into an insect mutualist (Koga et al., 2022). Additional virulence-associated traits warrant further investigation to better understand the ecological relevance and potential pathogenicity of *E. ruysiae* across different environments.

Comparative analysis of the antimicrobial susceptibility of our strains and *E. ruysiea* strain S1-IND-07-A from Campos-Madueno et al. (2023), revealed similar susceptibility to gentamicin, cefpodoxime, and trimethoprim-sulfamethoxazole., Importantly, the antimicrobial resistance findings in our study are not too concerning from a public health perspective because only the Bla-CTX-15 gene was detected in the genomes of the four isolates. However, all four strains exhibited resistance to clindamycin (lincosamide), erythromycin (macrolide), and metronidazole (nitroimidazole) in the *in vitro* disk diffusion AST profiles.

Pangenome analysis provided further insights into the genomic diversity and adaptive potential of *E. ruysiae*. The expansive accessory genome and the exponential pangenome evolution underscore the species’ genomic plasticity and its ability to acquire and integrate genetic materials from diverse sources, thereby shaping its evolutionary trajectory. This plasticity likely supports the organism’s capacity to live and adapt to various environments and niches. Additionally, phylogrouping based on core genome analysis allowed us to categorize *E. ruysiae* strains into two distinct phylogenetic groups, providing a useful framework for understanding their evolutionary relationships and tracking the geographical and ecological distribution of the species.

In conclusion, our study integrates culturomic, phenotypic, pangenomic, and phylogenetic analyses to provide a robust foundation for further exploration of the ecological, evolutionary, and clinical significance of the recently described species *E. ruysiae*.

## Data Availability

All four genomes sequenced for the study were submitted and referenced in the NCBI database (https://www.ncbi.nlm.nih.gov/bioproject/PRJNA1010808/) with the respective accession numbers *E. ruysiae* strain AB134 (JAVIWR000000000), *E. ruysiae* strain AB135 (JAVIWS000000000), *E. ruysiae* strain AB136 (JAVIWT000000000), and *E. ruysiae* strain AB137 (JAVIWU000000000).

## Footnotes

^1^ https://github.com/tseemann/mlst

^2^ https://github.com/tseemann/scapper

^3^ https://github.com/tseemann/abricate
