# Supplementary material for: Comparative genomic and phenotypic description of Escherichia ruysiae: a newly identified member of the gut microbiome of the domestic dog
Source: Front Microbiol. 2025 Apr 1;16:1558802. doi: 10.3389/fmicb.2025.1558802 (PMC11997573; doi:10.3389/fmicb.2025.1558802)
Supplement: Supplementary file 1 [file Table_1.docx]

**Supplementary Tables**

Table S1: Genomic accession numbers and links for the four new *E. ruysiae* strains

| **Isolate ID** | **Strain** | **BioProject** | **Accession** | **Taxid** | **WebLinks** |
| --- | --- | --- | --- | --- | --- |
| ABCS0134 | AB134 | PRJNA1010808 | JAVIWR | 2608867 | <https://www.ncbi.nlm.nih.gov/biosample/37199582> |
| ABCS0135 | AB135 | PRJNA1010808 | JAVIWS | 2608867 | <https://www.ncbi.nlm.nih.gov/biosample/37199583> |
| ABCS0136 | AB136 | PRJNA1010808 | JAVIWT | 2608867 | <https://www.ncbi.nlm.nih.gov/biosample/37199584> |
| ABCS0137 | AB137 | PRJNA1010808 | JAVIWU | 2608867 | <https://www.ncbi.nlm.nih.gov/biosample/37199585> |

Table S2: Genomic and metadata information for all *E. ruysiae* strains used in this study.

| **Species name & strain ID** | **Geographical location** | **host** | **Sample type** | **Sequencing type** | **Phylogroup** |
| --- | --- | --- | --- | --- | --- |
| *Escherichia ruysiae* strain S1-IND-07-A | Switzerland | *Homo sapiens* | feces | ST5792 | cladeIV |
| *Escherichia ruysiae* strain C61-1 | Japan | *Corvus macrorhynchos* | feces | ST3568 | cladeIII |
| *Escherichia ruysiae* strain C18-2 | United Kingdom | *Gallus gallus* | feces | ST9287 | cladeIII |
| *Escherichia ruysiae* strain C17-1 | United Kingdom | *Gallus gallus* | feces | ST4103 | cladeIV |
| *Escherichia ruysiae* strain C15-8 | United Kingdom | *Gallus gallus* | feces | ST2371 | cladeIII |
| *Escherichia ruysiae* strain C14-1 | United Kingdom | *Gallus gallus* | feces | ST11516 | cladeIV |
| *Escherichia ruysiae* strain C13-5 | United Kingdom | *Gallus gallus* | feces | ST6540 | cladeIII |
| *Escherichia ruysiae* strain C13-1 | United Kingdom | *Gallus gallus* | feces | ST11513 | cladeIII |
| *Escherichia ruysiae* strain C11-9 | United Kingdom | *Gallus gallus* | feces | ST6540 | cladeIII |
| *Escherichia ruysiae* strain C10-9 | United Kingdom | *Gallus gallus* | feces | ST11513 | cladeIII |
| *Escherichia ruysiae* strain C8-9 | United Kingdom | *Gallus gallus* | feces | ST9287 | cladeIII |
| *Escherichia ruysiae* strain C6-1 | United Kingdom | *Gallus gallus* | feces | ST3568 | cladeIII |
| *Escherichia ruysiae* strain C10-7 | United Kingdom | *Gallus gallus* | feces | ST4103 | cladeIV |
| *Escherichia ruysiae* strain OPT1704 | Netherlands | *Homo sapiens* | feces | ST6467 | cladeIV |
| *Escherichia ruysiae* strain AB134 | United States | *Canis lupus familiaris* | feces | ST9858 | cladeIV |
| *Escherichia ruysiae* strain AB135 | United States | *Canis lupus familiaris* | feces | ST9858 | cladeIV |
| *Escherichia ruysiae* strain AB136 | United States | *Canis lupus familiaris* | feces | ST9858 | cladeIV |
| *Escherichia ruysiae* strain AB137 | United States | *Canis lupus familiaris* | feces | ST9858 | cladeIV |

Table S3. Strain accession numbers of additional *Escherichia* strains used in this study

| **Organism Name** | **Strain** | **WGS project accession** | **Assembly Accession** |
| --- | --- | --- | --- |
| Escherichia ruysiae | C8-9 | JAINBF01 | GCF_019840505.1 |
| Escherichia ruysiae | C18-2 | JAIMZW01 | GCA_019839675.1 |
| Escherichia ruysiae | C6-1 | JAVHDE01 | GCF_036174885.1 |
| Escherichia ruysiae | C13-5 | JAINBN01 | GCF_019840805.1 |
| Escherichia ruysiae | C11-9 | JAVHDF01 | GCA_036181105.1 |
| Escherichia ruysiae | C15-8 | JAVHDF01 | GCF_036181105.1 |
| Escherichia ruysiae | C17-1 | JAINAL01 | GCA_019839665.1 |
| Escherichia ruysiae | C61-1 | JAINAL01 | GCF_019839665.1 |
| Escherichia ruysiae | C14-1 | JAINAT01 | GCA_019840085.1 |
| Escherichia ruysiae | C10-7 | JAINAT01 | GCF_019840085.1 |
| Escherichia ruysiae | S1-IND-07-A | JAINAA01 | GCF_019840185.1 |
| Escherichia ruysiae | C13-1 | JAIMZX01 | GCF_019839605.1 |
| Escherichia ruysiae | C10-9 | JAMSJK01 | GCA_024733345.1 |
| Escherichia ruysiae | OPT1704 | CABVLQ01 | GCA_902498915.1 |
| Escherichia albertii | 167 | CP070290.2 | GCA_016904755.2 |
| Escherichia coli | K-12 (NC 000913) | NC_000913.3 | GCA_000005845.2 |
| Escherichia fergusonii | FDAARGOS 1499 | NZ_CP083638.1 | GCA_020097475.1 |
| Escherichia marmotae | H1-003-0086-C-F | CACSXJ02 | GCA_902709585.2 |
| Escherichia whittamii | C2-3 | JAINCF01 | GCF_020283705.1 |
