# Supplementary figures and images for: Comparative genomic and phenotypic description of Escherichia ruysiae: a newly identified member of the gut microbiome of the domestic dog
Source: Front Microbiol. 2025 Apr 1;16:1558802. doi: 10.3389/fmicb.2025.1558802 (PMC11997573; doi:10.3389/fmicb.2025.1558802)

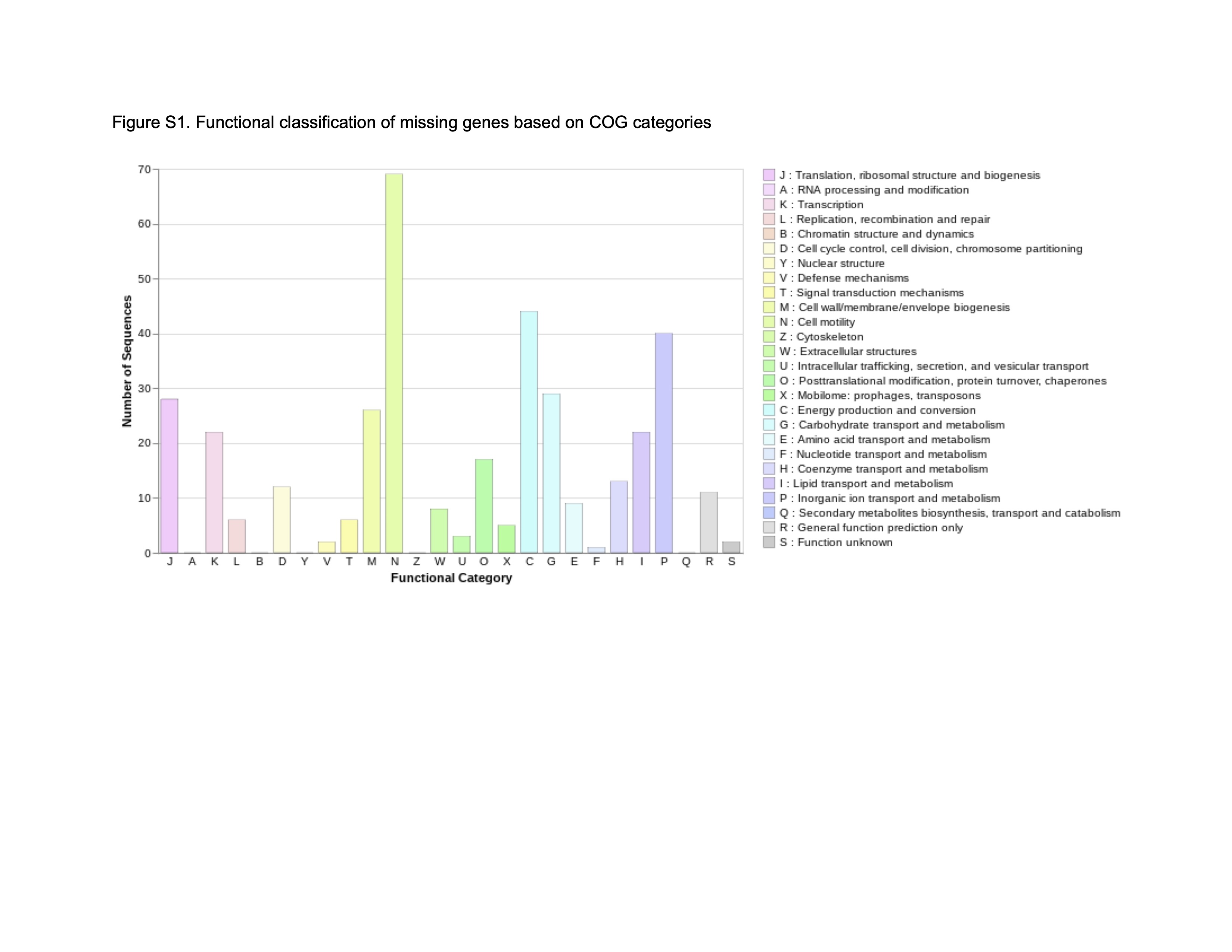

Supplement: Supplementary file 3 [file Image_1.jpeg]
